# Lemierre Syndrome Complicating Deep Neck Infection and Descending Mediastinitis Secondary to Methicillin resistant Staphylococcus aureus (MRSA) Infection

**DOI:** 10.12669/pjms.38.5.5442

**Published:** 2022

**Authors:** Abdulqader Al-Hebshi, Hind Alharbi, Rayan Karbouji, Ghaya Al Qurainees, Ahmad Alkurdi

**Affiliations:** 1 Abdulqader Al-Hebshi, Department of Pediatric, Prince Mohammad bin Abdul Aziz Hospital, Ministry of National Guard-Health Affairs, Madina, Saudi Arabia; 2 Hind Alharbi, Department of Pediatric, Prince Mohammad bin Abdul Aziz Hospital, Ministry of National Guard-Health Affairs, Madina, Saudi Arabia; 3 Rayan Karbouji, Department of Pediatric, Prince Mohammad bin Abdul Aziz Hospital, Ministry of National Guard-Health Affairs, Madina, Saudi Arabia; 4 Ghaya Al Qurainees, Department of Pediatric, Prince Mohammad bin Abdul Aziz Hospital, Ministry of National Guard-Health Affairs, Madina, Saudi Arabia; 5 Ahmad Alkurdi, Division of ENT, Department of Surgery, Prince Mohammad bin Abdul Aziz Hospital, Ministry of National Guard-Health Affairs, Madina, Saudi Arabia

**Keywords:** Lemierre syndrome, Jugular vein, Thrombophlebitis, MRSA

## Abstract

Lemierre syndrome (LS) is an oropharyngeal infection, followed by bacteremia, thrombosis of the internal jugular vein and subsequent hematogenic metastasis via septic emboli. We report a case of Lemierre syndrome complicated by descending necrotizing mediastinitis due to a Methicillin-resistant Staphylococcus aureus (MRSA) infection in a 5-month-old Saudi boy.

## INTRODUCTION

Lemierre syndrome (LS), known as post anginal septicemia, was first reported in 1936 by Dr. Lemierre.^1^ In his original publication 18 of 20 patients died, indicating a high mortality rate (90%). It was mostly caused by Fusobacterium necrophorum, although many bacteria have since been implicated.^2^ With the introduction of antibiotics, Lemierre syndrome became the “forgotten disease” as its incidence and mortality decreased dramatically.^3^,^4^ Nevertheless, an increase in LS cases has been reported over the past decade, probably due to increased antibiotic resistance or a modification in the prescription.^5^

Patients primarily present with oropharyngeal infection, accompanied by parapharyngeal and internal jugular vein (IJV) involvement. Septic emboli cause the spread of the infection, sepsis and metastatic lesions emerge, resulting in serious morbidity and fatality.^6^,^7^ Despite the fact that 85% of LS cases originated from pharyngitis or tonsillitis, 3% and 2% of LS cases are caused by mastoiditis and odontogenic infections, respectively.^8^,^9^ The age of the disease onset can vary between two months and 79 years. The youngest case, recently reported, involved a 5-week-old female with peritonsillar/parapharyngeal derived LS.^10^,^11^ We report a case of Lemierre syndrome complicated with descending necrotizing mediastinitis due to a Methicillin-resistant Staphylococcus aureus (MRSA) infection in a 5-month-old boy in Saudi Arabia.

### Case Presentation:

A previously healthy 5-month-old Saudi boy presented at the Emergency Department (ED) with a history of fever, poor oral intake, and swelling of the neck two days prior to ED presentation. Physical examination revealed a temperature of 39ºC, the respiratory rate was 54 breaths/minute, the oxygen saturation 99% in room air, the heart rate 190 beats/minute, and the blood pressure 111/60 mmHg. The examination of his chest, cardiovascular and abdominal systems were unremarkable. He had a hyperemic soft palate (throat difficult to assess), otherwise a clear oral cavity with no wounds, ulcer or discoloration. The ears were not inflamed. Cervical swelling (3cm x 5cm) occurred on the left side, it was tense and firm, non-mobile, warm, red and tender to palpation, with no obvious site of wound or pus. No other lymph nodes were palpable. The rest of the systemic examination was unremarkable. The blood investigation indicated a normal white blood cell (WBC) count of 7.5 x10 ^9/L (differential with 69.6% neutrophils, 0.04% eosinophils, 21.40% lymphocytes, 8.4% monocytes and 0.11% basophils), CRP 244 mg/L and ESR 69 mm/hr. The COVID-19 test was negative.

He was admitted to a general pediatric ward, diagnosed with left cervical lymphadenitis with adjacent cellulitis and cefazolin was prescribed. One day after admission, the patient improved, the fever became less frequent and of low a grade. The lymph node decreased in size and the warmth, redness and tenderness disappeared.

On Day 3 post-admission, although the patient become afebrile, the lymph node decreased in size and the cellulitis resolved, due to poor oral intake with intermittent stridor and hoarseness with subcostal retraction during crying, the patient was transferred to PICU for close monitoring and an urgent otorhinolaryngologist consultation was done. A complete head and neck examination with a flexible nasolaryngoscopy showed plugging in the posterior pharyngeal wall with mild supraglottic edema.

**Fig.1:**
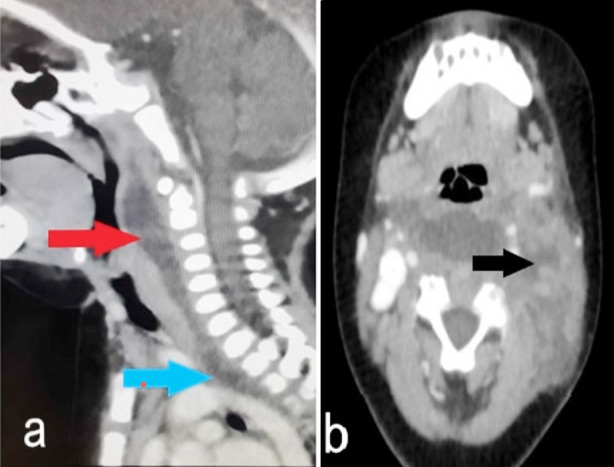
CT scan of the neck with contrast. (a) Multi loculated large retropharyngeal access collection seen extended into the parapharyngeal space more toward left side involving left posterior triangle (Red arrow). The abscess extending into the anterior and posterior mediastinum (Blue arrow). (b) Non-visualised left deep jugular vein that indicate presence of thrombus (black arrow).

A neck and brain computed tomography (CT) with contrast (Fig.1), showed a large retropharyngeal and left para-pharyngeal abscess extending into the anterior and posterior mediastinum. The CT ruled out any obvious intracranial extension. A thrombus was reported in the left IJV, which was compatible with the diagnosis of Lemierre syndrome. The antibiotic was upgraded to vancomycin and meropenem. He was started on enoxaparin 1 mg/kg/day/ twice per day as a therapeutic dose for the left IJV thrombosis.

**Fig.2:**
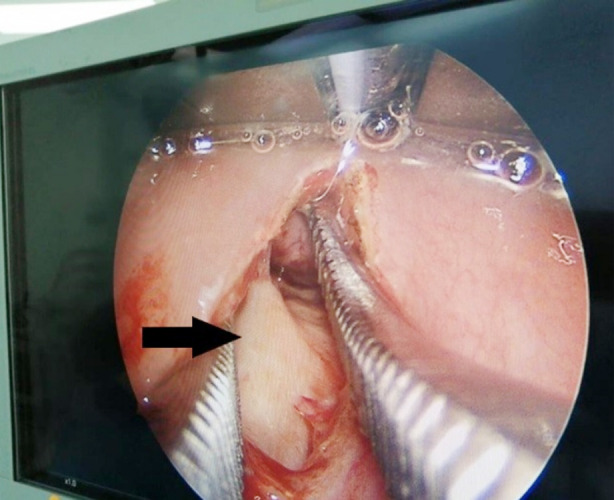
An intra-operation photo demonstrating the abscess cavity, the posterior pharyngeal wall and the pre-vertebral fascia (black arrow).

The patient was taken to the operation theater for an emergency drainage of the deep neck infection abscess. Trans-oral retropharyngeal abscess drainage was done with the evacuation of a large amount of pure pus (Fig.2). A drainage sample was sent for culture confirming a methicillin-resistant Staphylococcus aureus (MRSA). The patient continued on vancomycin and the meropenem was discontinued as we had a definitive organism. One week after the incision and drainage, laboratory and radiological investigations indicated a slow radiological improvement with a high inflammatory marker. We considered the infection as polymicrobial and the meropenem was resumed. The vancomycin was changed to linezolid because linezolid is as effective as vancomycin for the treatment of MRSA infections, and it may be more effective than vancomycin in achieving microbiological eradication.

The patient’s clinical condition improved, and his laboratory and radiographic findings improved as well, so he was sent home to continue his treatment, which included six weeks of linezolid and Flagyl and three months of enoxaparin therapy. The patient was followed up at a pediatric infectious disease clinic every two weeks for an additional three months after discharge, where he showed complete resolution of the neck lesion as well as normalization of all inflammatory markers.

## DISCUSSION

This case described a 5-month-old patient. Although LS affects mainly young adults and adolescents, many young cases were reported with different causative agents, such as a 2-month-old male with otomastoiditis, two boys, 2.4-month-old and 5-month-old, with otitis media, and a 5-week-old boy (the youngest case) with a peritonsillar/parapharyngeal derived LS.^10^,^12^-^14^ Previous evidence indicated that males were at a two-fold higher risk of developing Lemierre syndrome.^5^

The culture from the abscess aspiration showed gram-positive cocci in clusters confirming MRSA infection. Chirinos et al. reported that in cases with a positive culture, Fusobacterium necrophorum was the most common causative agent (81.7%), with a small proportion (5.5%) positive for bacteria other than Fusobacterium necrophorum, such as Staphylococcus aureus, as in our study.^2^ On admission, the infant showed non-specific symptoms, including fever and neck swelling. Lemierre highlighted that “The syndrome is so characteristic that mistake is impossible”.^1^ However, the lack of professional awareness and the rarity of LS cases usually cause a delayed diagnosis, and most patients present with metastatic complications by the time of a definitive diagnosis.^2^

In the present case, the culture positive MRSA, the radiographic evaluation of the neck with ultrasonography, CT scan, and MRI confirming IJV thrombosis and metastatic lesions in lungs, were the clues to diagnose LS. Riordan et al. reported that the essential diagnostic criteria defining LS were: a) Oropharyngeal infection in the last four weeks, b) clinical manifestations of metastatic lesions in lungs and/or another remote site, and c) Evidence of IJV thrombophlebitis or confirmation of Fusobacterium necrophorum or Fusobacterium sp. with a blood culture of a normally sterile site.^15^ The flora of deep neck space infections might include aerobic and anaerobic bacteria with anaerobes predominant, other than Fusobacterium spp. such as Staphylococcus aureus in our case.^16^ Overall, clinical suspicion should be high when a patient is admitted with any head and neck symptoms in addition to signs of IJV thrombophlebitis, sepsis, or systemic organ failure via septic emboli.

As therapeutic management, we chose a specific combination of aggressive antimicrobial treatment with anticoagulation and abscess drainage. The administration of anticoagulants in LS patients is still controversial.^6^,^17^ Some authors claim its role as increasing the rate of thrombophlebitis resolution with clot destruction, while others suggest a higher risk of hemorrhage and the extent of infection secondary to thrombolysis.^5^ Ultimately, anticoagulation therapy is related to personal preference and institution policies.^17^

## CONCLUSION

This is the first case in Saudi Arabia describing a 5-month-old male with a deep neck infection, and descending mediastinitis secondary to MRSA infection derived LS. Morbidity and death can be avoided with early diagnosis; however, awareness of LS and a high degree of suspicion are required.

### Authors’ Contribution:

**AA** wrote the manuscript and responsible for the integrity and accuracy of the study.

**GA and AK** helped in the writing and designing of the manuscript.

**HA, RK** collected the data and participated in the editing and the coordination.

**AA, AK** supervised and reviewed the final manuscript. **AA** is the project leader.

All authors read and approved the final version.
